# Protein loop structure prediction by community-based deep learning and its application to antibody CDR H3 loop modeling

**DOI:** 10.1371/journal.pcbi.1012239

**Published:** 2024-06-24

**Authors:** Hyeonuk Woo, Yubeen Kim, Chaok Seok

**Affiliations:** 1 Department of Chemistry, Seoul National University, Seoul, Republic of Korea; 2 Galux Inc. Seoul, Republic of Korea; Hebrew University of Jerusalem, ISRAEL

## Abstract

As of now, more than 60 years have passed since the first determination of protein structures through crystallography, and a significant portion of protein structures can be predicted by computers. This is due to the groundbreaking enhancement in protein structure prediction achieved through neural network training utilizing extensive sequence and structure data. However, substantial challenges persist in structure prediction due to limited data availability, with antibody structure prediction standing as one such challenge. In this paper, we propose a novel neural network architecture that effectively enables structure prediction by reflecting the inherent combinatorial nature involved in protein structure formation. The core idea of this neural network architecture is not solely to track and generate a single structure but rather to form a community of multiple structures and pursue accurate structure prediction by exchanging information among community members. Applying this concept to antibody CDR H3 loop structure prediction resulted in improved structure sampling. Such an approach could be applied in the structural and functional studies of proteins, particularly in exploring various physiological processes mediated by loops. Moreover, it holds potential in addressing various other types of combinatorial structure prediction and design problems.

## Author summary

In this paper, we propose a new architecture that aims to improve upon protein structure prediction algorithms like AlphaFold or RoseTTAFold by considering the combinatorial nature of protein structure formation. Such an architecture, reflecting the physical principles of nature, is expected to yield beneficial results, particularly in scenarios with limited structure and sequence information. Named ComMat, this architecture does not focus on a single structure but rather on a set of multiple structures—a community—simultaneously. In this process, combinatorial exploration of protein structure is encouraged through information exchange among community members. ComMat is an instance that integrates this idea within the structure module of AlphaFold. Applying ComMat to antibody CDR H3 loop structure prediction yielded outstanding results in structure sampling and prediction when tested on the IgFold set and compared with IgFold and AlphaFold-Multimer. It confirmed that improved structure sampling stems from effective structural exploration. The proposed concept here could potentially be used in the development of various other combinatorial protein structure prediction and protein design methods.

## 1. Introduction

Thanks to the remarkable achievements of AlphaFold2 [1] and RoseTTAFold [2], protein structure prediction has emerged as a pivotal tool in numerous biological research domains. These advancements signal a new era in biology and medicine [3–5]. However, a notable limitation of these state-of-the-art protein structure prediction methods is their heavy reliance on sequence co-variation information of proteins evolutionarily related to the target protein [6–8].

While there is evidence suggesting that these methods have, to some extent, learned the principles of protein folding and binding [9–11], accurate structure prediction becomes considerably challenging when there is insufficient structural and sequence information. One prominent example of this challenge pertains to predicting antibody-antigen complex structures. There exists a lack of sequence evolutionary information that reveals deep sequence correlations specific to a particular antibody-antigen complex. Furthermore, the protein structure database used for training deep neural networks contains significantly more information about secondary structures than loop structures, such as those found in antibodies.

Hence, there is a demand for new methods capable of accurately predicting protein structures even when lacking structural and sequence information from evolutionarily related proteins. To achieve precise predictions with limited information about a particular problem, the architecture of the prediction model should effectively mirror the nature of that problem. In this study, we propose a structure prediction architecture that effectively captures the combinatorial nature of protein structure formation. This method, referred to as ‘community maturation’ or ComMat, demonstrates successful performance in predicting antibody CDR H3 loop structures.

The 3D structure of a protein consists of individual amino acid structures that collectively form an assembly, with each assuming an optimal structure within its spatially proximate structural environment. Conflicting optimal structures for different amino acids lead to a compromise, resulting in an overall optimal state. This overall protein structure is attained through an optimal combination of the component amino acid structures, even if some of these components may be suboptimal. Classic structure optimization methods leveraging this combinatorial nature include genetic algorithms [12] and conformational space annealing [13–18]. Instead of focusing solely on a single structure, these methods optimize structures by evolving an ensemble of multiple structures. In this process, they attempt effective predictions by exploiting the combinatorial nature through the exchange of information among various pairs of ensemble members.

Our ComMat architecture integrates the concept of structural ensemble evolution into the AlphaFold structure module [1]. Here, the structural ensemble is denoted as a ’community,’ where the community size indicates the number of structures within the ensemble. The process of ensemble evolution is referred to as ‘community maturation.’ Compared to a typical structure prediction neural network architecture that tracks representations for a single structure, we demonstrate that the community maturation method becomes increasingly effective in sampling protein loop structures as the community size increases, resulting in more accurate structure prediction.

To illustrate, we trained the ComMat model to sample loop structures of proteins with experimentally resolved structures, focusing on antibody CDR H3 loops. The objective was to obtain at least one community member representing a loop structure as close as possible to the known experimental structure. Using ComMat to sample the CDR H3 loop structures of antibodies, starting from the antibody framework structures predicted with IgFold [19], and evaluating them with the accuracy estimate of ComMat resulted in improved loop structure prediction compared to IgFold. Specifically, the percentage of accurately predicted loop structures within a 2 Å threshold increased from 33.5% to 39.6% when tested on the IgFold set [19]. Moreover, achieving a success rate of sampling loop structures within 2 Å at 60.9% with a community size of 32 implies the potential for even greater accuracy in loop structure prediction by employing a more advanced scoring method.

The significance of this study, which introduces a new neural network architecture focusing on antibody CDR H3 loop structures, extends beyond the critical role antibodies play as therapeutic modalities. It also holds relevance for research on protein structure and function involved in various other essential physiological processes facilitated through loop-mediated interactions, such as T-cell receptors or G-protein-coupled receptors.

## 2. Results and discussion

### 2.1. A Community-Based Neural Network Architecture: ComMat for Protein Loop Structure Prediction

**Fig 1:**
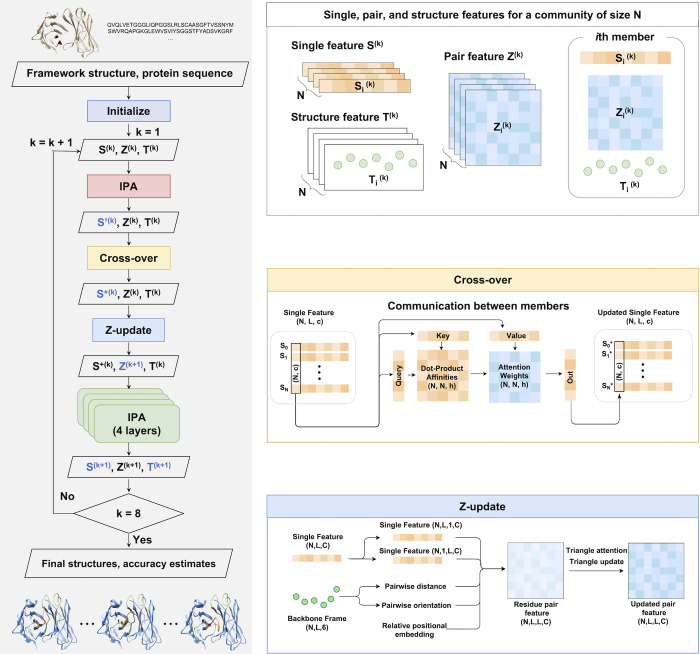
The overall workflow of ComMat. At each cycle, a community of size *N*, represented by single, pair, and structure features, progresses through updates facilitated by communication via cross-over and pair feature update.

The ComMat neural network architecture was developed to harness the combinatorial nature of protein structures effectively. It achieves this by incorporating the concept of structural ensemble evolution into the AlphaFold’s structure module [1]. The workflow of the architecture is depicted in **Fig 1**.

In line with the structure module of AlphaFold, we employed three types of features–*S* (single feature, with 128 channels for each residue), *Z* (pair feature, with 96 channels for each residue pair), and *T* (structure feature, representing 6 spatial translation and rotation degrees of freedom for each residue gas)–to represent a single protein structure. However, unlike evolving representations for a single structure, ComMat evolves representations for a community of size *N*. This community is represented by single features $S^{\left(k\right)}=(S_{1}^{\left(k\right)},S_{2}^{\left(k\right)},\cdots ,S_{N}^{\left(k\right)})$, pair features $Z^{\left(k\right)}=(Z_{1}^{\left(k\right)},Z_{2}^{\left(k\right)},\cdots ,Z_{N}^{\left(k\right)})$, and structure features $T^{\left(k\right)}=(T_{1}^{\left(k\right)},T_{2}^{\left(k\right)},\cdots ,T_{N}^{\left(k\right)})$. Here, the superscript indicates the cycle number, and the subscript the community member index. For loop structure prediction, eight cycles were employed using identical network parameters. The same IPA (Invariant Point Attention) architecture as AlphaFold was utilized to update single and structure features based on single and pair features.

In contrast to the fixed pair feature Z in the AlphaFold structure module, ComMat updates the community pair features ***Z***^(*k*)^ within each cycle through ’Z-update’ [**Fig 1**, Materials and Methods 3.2.3], following the exchange of single feature information among pairs of community members via ’Cross-over’ using community-wide attention [**Fig 1**, Materials and Methods 3.2.2]. This cross-over operation occurs for the updated single features ***S***′^(*k*)^ from the preceding single and pair features with a single layer of IPA. Subsequently, the structure features ***T***^(*k*)^ are updated in each cycle by four layers of IPA after the single feature cross-over and pair feature update.

The initialization of ComMat involves generating *N* initial loop structures randomly positioned between the two stem residues within the input framework structure [Materials and Methods 3.2.1]. The procedures for generating initial single and pair features are also detailed in Materials and Methods.

Finally, the prediction accuracy for each community member is estimated from the final single features as an average of the predicted Local Distance Difference Test (pLDDT) [20] for each residue. This accuracy estimate was then used to rank the sampled loop structures. Subsequently, the final antibody structure underwent geometry optimization with GALAXY energy [14] to enhance its physical realism. As an alternative ranking method, we also experimented with AF2Rank [9], using each sampled structure as input to the AlphaFold-Multimer 2.2 model 5.

The ComMat architecture serves as an illustration of incorporating the conformational sampling idea, stemming from classical ensemble-based structure optimization methods. It is anticipated that the effective communication between community members for complex structure prediction problems will facilitate a more efficient exploration of the conformational space compared to models that sample only one structure at a time. A downside of ComMat is its increased computer memory requirement in implementation, but advancements in hardware technologies would enable more efficient exploration of such methods.

### 2.2. Performance of ComMat in Antibody CDR H3 Loop Structure Prediction: Comparison of Models with and without Cross-over

The effectiveness of ComMat in predicting antibody CDR H3 loop structures, crucial in antigen binding but challenging to predict due to their wide variability [21–24], is demonstrated herein. The ComMat concept introduced here is directly applicable to diverse structure prediction tasks, including full structure prediction, structure refinement, and docking.

To assess ComMat’s performance, we employed a benchmark set of 197 antibodies, previously introduced in evaluating the IgFold antibody structure prediction model [19]. This set includes experimentally resolved structures released post-July 1, 2021. Therefore, in training ComMat for loop modeling, we constructed a new dataset comprising experimental structures released before this date [Materials and Methods 3.1].

In this test, the antibody framework structure generated by IgFold was used as input, and the H3 loop structure was reconstructed without utilizing any information from the input loop structure. To account for the inherent randomness in ComMat due to initial randomization, the performance evaluation was conducted by averaging the results obtained from five trials.

**Fig 2:**
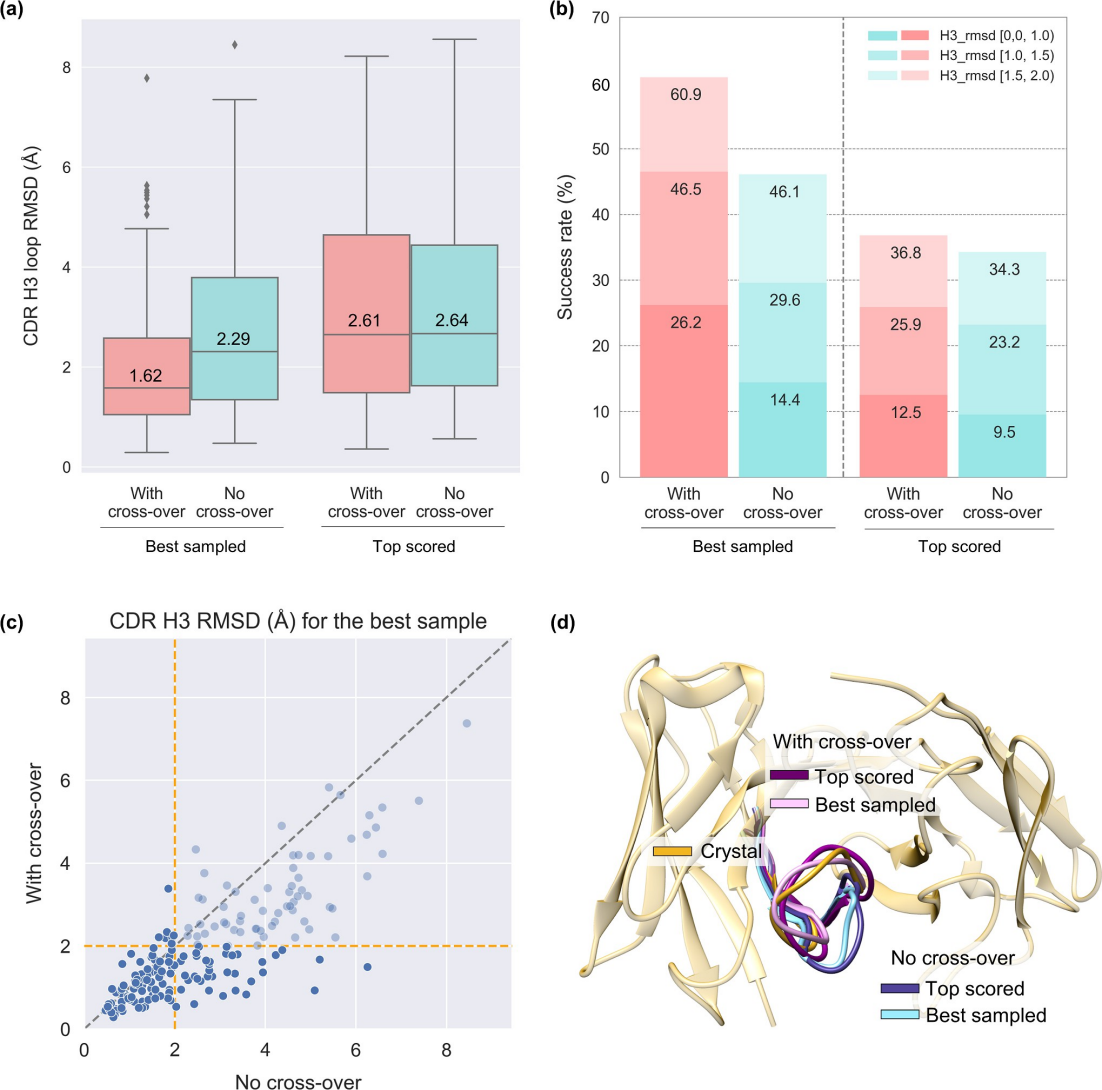
Performance comparison between ’With cross-over’ and ’No cross-over’ prediction setups on the IgFold set. (a) Structure prediction errors measured by loop RMSD for best sampled and top scored models with and without cross-over (*N* = 32). Box plots display the median, interquartile range (IQR) bounds, whisker length of 1.5 ✕ IQR, and outliers beyond the 1.5 ✕ IQR range. (b) Success rates of best sampled and top scored structures with and without cross-over. (c) Best RMSD obtained by the two algorithms for individual targets. (d) Example case (PDB ID 7WPH) where best sampled and top scored models with cross-over (RMSD = 1.34 Å and 0.93 Å, respectively) outperform those without cross-over (3.06 Å and 2.75 Å, respectively).

We trained ComMat with various community sizes *N* to assess its efficacy, as elaborated later in Results and Discussion 2.5. In **Fig 2**, the H3 loop structure prediction results obtained using a single inference of ComMat trained with *N* = 32 (referred to as ’With cross-over’) are compared with 32 independent inferences of ComMat trained with *N* = 1 (referred to as ’No cross-over’). Loop structure prediction errors were measured using loop RMSD, defined as the root-mean-square deviation of backbone heavy atom (N, Cα, C) coordinates from the experimental loop structure. This measurement was taken after aligning the framework Cα atoms, with the non-loop framework region identified by the Chothia numbering scheme.

Results from the ’No cross-over’ setup establish a baseline performance where no information exchange occurs between structure samples. As depicted in **Fig 2A**, the median RMSD of the best sampled structure among 32 loop structures generated by ’No cross-over’ (*N* = 1) for the IgFold set is 2.29 Å. In contrast, the median RMSD of the best sampled structure by a single inference of the ’With cross-over’ setup (*N* = 32) is 1.62 Å, highlighting a substantial improvement in loop structure sampling through community maturation.

When these sampled structures were scored using the ComMat accuracy estimate, the median RMSD of the top scored structures was 2.61 Å and 2.64 Å with and without cross-over, respectively. This suggests that while cross-over explores more diverse structures, training on the sampling performance does not compromise structure prediction performance. Integrating a separate scoring scheme could further enhance structure prediction.

A consistent trend of enhanced performance with cross-over was observed in the percentage of cases predicted within RMSD cut-offs, as illustrated in **Fig 2B**. The success rate of sampling loop structures within 2 Å was 60.9% and 46.1% with and without cross-over, respectively. After scoring with the accuracy estimate, the success rate of loop structure prediction within 2 Å was 36.8% and 34.3% with and without cross-over, respectively.

Analyzing individual test cases in **Fig 2C** revealed improved sampling performance in loop RMSD in 161 of 197 cases with cross-over. Moreover, cross-over rescued 32 of 103 cases predicted with RMSD > 2 Å without cross-over, achieving RMSD < 2 Å. The source data is provided in **S3 Table**.

**Fig 2D** presents a case (PDB ID 7WPH, H3 loop length = 12, the maximum sequence identity with a training set antibody = 50%) where cross-over led to improved loop structure sampling and prediction within RMSD 1.5 Å compared to a prediction with RMSD > 2 Å without cross-over.

In summary, the above results confirm that ComMat substantially enhances loop structure sampling performance through cross-over among community members when applied to antibody CDR H3 loop structure prediction from predicted antibody framework structures by IgFold. The robustness in predicting H3 loop structures for “model” antibody structures stems from the training strategy involving perturbed antibody structures [Materials and Methods 3.1]. Additionally, it was observed that loop structure prediction performance improves with community exchange when ranking the sampled structures using the ComMat accuracy estimate. Further enhancements in structure prediction accuracy are expected by employing a separately trained scoring model.

### 2.3. Performance of ComMat in Antibody CDR H3 Loop Structure Prediction: Comparison with Other Available Methods

The antibody CDR H3 loop structure prediction results of ComMat were analyzed by comparing them with predictions from deep learning methods IgFold [19], AlphaFold-Multimer 2.2 and 2.3 [25], ImmueBuilder [24], EquiFold [26], and a traditional physics-based model, RosettaAntibody [27]. These comparisons focused on sampling performance in **Table 1** and structure prediction performance in **Table 2**. The raw data used to generate these tables are provided as Supplementary Materials (**S5 Table** and https://github.com/seoklab/ComMat).

**Table 1:** Median CDR H3 loop RMSD of the best sampled structures for the test complexes in the IgFold set for different methods.

Test set^a^		ComMat *N* = 1, 4, 32^b^	IgFold *N* = 4^c^	Immune Builder *N* = 4^d^	EquiFold *N* = 1^e^	Rosetta Antibody *N* = 1, 32^f^	AFM2.2–1 *N* = 1, 32^g^	AFM2.3–1 *N* = 1, 32^g^
After ‘21.07.01	100% (197)	2.70, 2.20**, 1.63**	2.36	-	2.25	-	2.27, 1.78	-
	95% (170)	2.84, 2.42, **1.75**	2.53	-	2.44	-	2.34, 1.84	-
After ‘21.08.01	100% (177)	2.67, 2.18, **1.64**	2.29	1.88	2.43	-	2.24, 1.76	-
	95% (153)	2.83, 2.40, **1.78**	2.52	1.92	2.50	-	2.33, 1.78	-
After ‘21.10.01	100% (153)	2.69, 2.20, 1.65	2.36	2.02	2.45	-	2.18, 1.63	2.16, **1.59**
	95% (133)	2.86, 2.47, 1.79	2.54	2.17	2.63	-	2.26, 1.76	2.25, **1.65**
Rosetta Antibody	100% (176)	2.64, 2.15, **1.63**	2.33	-	2.12	3.62, 2.19	2.23, 1.77	-
	95% (122)	3.11, 2.65, **1.72**	2.52	-	2.34	3.80, 2.30	2.34, 1.81	-

^a^Different test sets were curated to compare ComMat with various methods, each associated with different training database dates or where results were unavailable for some targets (e.g., Rosetta Antibody, for which results for 21 complexes were not obtained due to a runtime error). The first six sets were curated based on the published date and the maximum H3 loop sequence identity of a test complex to the training set. The resulting number of complexes is shown in parentheses.

^b^The ComMat model, trained with different seed sizes (*N* = 1, 4, and 32), was used for inference with the corresponding *N*. The average of five inferences were used.

^c^Sampling results for the four IgFold models with different parameters are presented. The best sample was selected for each complex. Complexes with PDB IDs 7WKX and 7X9E were not included due to runtime failures during refinement.

^d^Sampling results for the four ImmuneBuilder models are presented. The best sample was selected for each complex.

^e^EquiFold can generate only single models.

^f^RosettaAntibody was run to generate a total of 2,800 structures for each complex and *N* = 1 and 32 structures were chosen based on the Rosetta energy.

^g^Sampling results for AlphaFold-Multimer model 1 are presented. For *N* = 1, an average over 32 different runs is reported.

**Table 2:** Median CDR H3 loop RMSD of the top-ranking structures for the test complexes in the IgFold set for different methods.

Test set^a^		ComMat^b^	ComMat & AF2Rank^c^	IgFold^d^	Immune Builder	EquiFold	Rosetta Antibody^e^	AFM2.2^f^	AFM2.3^f^
After ‘21.07.01	100% (197)	2.64	2.43	2.73	-	**2.25**	-	**2.25**	-
	95% (170)	2.84	2.55	2.76	-	2.44	-	**2.31**	-
After ‘21.08.01	100% (177)	2.62	2.45	2.74	**2.21**	2.43	-	**2.21**	-
	95% (153)	2.79	2.56	2.76	2.37	2.50	-	**2.28**	-
After ‘21.10.01	100% (157)	2.66	2.42	2.76	2.37	2.45	-	**2.18**	2.20
	95% (133)	2.85	2.53	2.86	2.49	2.63	-	**2.25**	2.29
Rosetta Antibody	100% (176)	2.57	2.40	2.71	-	**2.12**	3.62	2.22	-
	95% (152)	2.77	2.50	2.75	-	2.34	3.80	**2.31**	-

^a^Different test sets were curated to compare ComMat with various methods, each associated with different training database dates or where results were unavailable for some targets (e.g., Rosetta Antibody, for which results for 21 complexes were not obtained due to a runtime error). The first six sets were curated based on the published date and the maximum H3 loop sequence identity of a test complex to the training set. The resulting number of complexes is shown in parentheses.

^b^Prediction results were obtained by sampling with *N* = 32 and ranked using the ComMat accuracy estimate.

^c^Prediction results were obtained by sampling with *N* = 32 and ranked using AF2Rank.

^d^Complexes with PDB IDs 7WKX and 7X9E were not included due to runtime failures during refinement.

^e^RosettaAntibody was run to generate a total of 2,800 structures for each complex and the final structure was chosen based on the Rosetta energy.

^f^Prediction results for AlphaFold-Multimer using all five models are presented. An average over 32 independent runs is reported.

Given that the current implementation of ComMat predicts only loop structures within a provided framework structure, our focus was solely on comparing the accuracy of loop structure predictions. For this comparison, we re-ran each method to generate different numbers (*N*) of structures and evaluated loop RMSDs of the structures ourselves. The comparison of different methods involved different subsets of the IgFold test set [19], to ensure no overlap with the training data of each method. Additionally, a subset with a maximum H3 loop sequence identity of 95% to the training set was employed to examine the dependency on sequence similarity. Detailed data for each target in the IgFold set is provided in **S4 Table**.

As shown in **Table 1**, ComMat, with *N* = 32, was capable of sampling loop structures with a median RMSD of 1.63 Å on the IgFold set (comprising 197 antibodies), whereas IgFold’s four models achieved a median RMSD of 2.36 Å within the same antibody framework structures. Although IgFold does not allow for additional sampling, AlphaFold-Multimer was evaluated for its ability to sample additional structures using different random seeds. Specifically, AlphaFold-Multimer 2.2 model 1 showed a median loop RMSD of 1.78 Å when generating 32 structures on the same IgFold set. AlphaFold-Multimer 2.3 model 1 demonstrated the highest sampling performance with 32 samples. Given the high performance of AlphaFold-Multimer with *N* = 1, which utilizes multiple sequence alignment (MSA) and template information processed in the AlphaFold Evoformer, the performance of ComMat—relying solely on the AlphaFold structural module without Evoformer—highlights the effectiveness of the community maturation architecture.

Like IgFold, ImmuneBuilder does not allow additional sampling. Its four models demonstrated high sampling performance with a median RMSD of 1.88 Å for 177 antibodies in the IgFold set and 2.02 Å for the 153 antibodies published after October 1, 2021. RosettaAntibody with *N* = 32 showed slightly better sampling performance (2.19 Å) than the four models of IgFold (2.33 Å) on the 176 antibodies for which RosettaAntibody successfully produced results. The source data for the above results are provided in **S6 Table**.

The structure prediction performance of ComMat (*N* = 32) after ranking sampled structures using the ComMat accuracy estimate and AF2Rank [9] is presented in **Table 2**, alongside the prediction results of other methods. ComMat demonstrates improved H3 loop structure prediction performance, achieving RMSDs of 2.64 Å and 2.43 Å with the ComMat accuracy estimate and AF2Rank, respectively, compared to IgFold’s 2.73 Å for the 197 antibodies in the IgFold set. Although ImmuneBuilder [24] and EquiFold [26] show higher overall performance, their RMSD values exhibit a greater dependence on the test set, leading to poorer performance with more recent test data. For the most recent dataset of 157 antibodies (published after October 1, 2021) in **Table 2**, the performance gap is very small: 2.42 Å, 2.37 Å, and 2.45 Å for ComMat with AF2Rank, ImmuneBuilder, and EquiFold, respectively. AlphaFold-Multimer 2.2, which uses MSA and templates, showed the highest overall performance, while RosettaAntibody displayed the lowest in **Table 2**. The source data for these results are provided in **S7 Table**.

**Fig 3:**
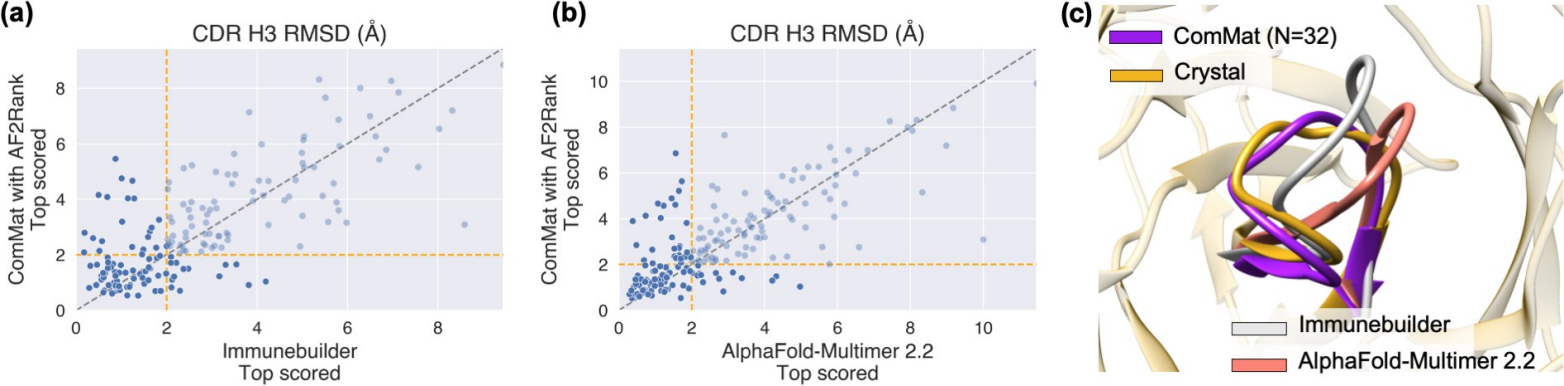
Comparison of predictions by ComMat with AF2Rank (*N* = 32) against (a) ImmuneBuilder and (b) AlphaFold-Multimer 2.2 for individual prediction targets, illustrating that different methods excel with different targets. (c) An example case (PDB ID: 7S0B) in which ComMat achieved higher prediction accuracy compared to both ImmuneBuilder and AlphaFold-Multimer 2.2.

**Fig 3** compares the prediction performance of ComMat with AF2Rank (*N* = 32) against ImmuneBuilder and AlphaFold-Multimer 2.2 for individual targets. The source data are provided in **S7 Table**. ComMat with AF2Rank demonstrates comparable overall performance across various targets, serving as an orthogonal approach to the others. An example case where ComMat outperforms the other two methods is shown in **Fig 3**C (PDB ID: 7S0B, H3 loop length = 12, maximum sequence identity to the training set = 58%), where it achieved an RMSD of 1.39 Å, improving from 2.37 Å and 3.79 Å for ImmuneBuilder and AlphaFold-Multimer 2.2, respectively.

### 2.4. Analysis of the Sampling Trajectory by ComMat with and without Cross-over

**Fig 4:**
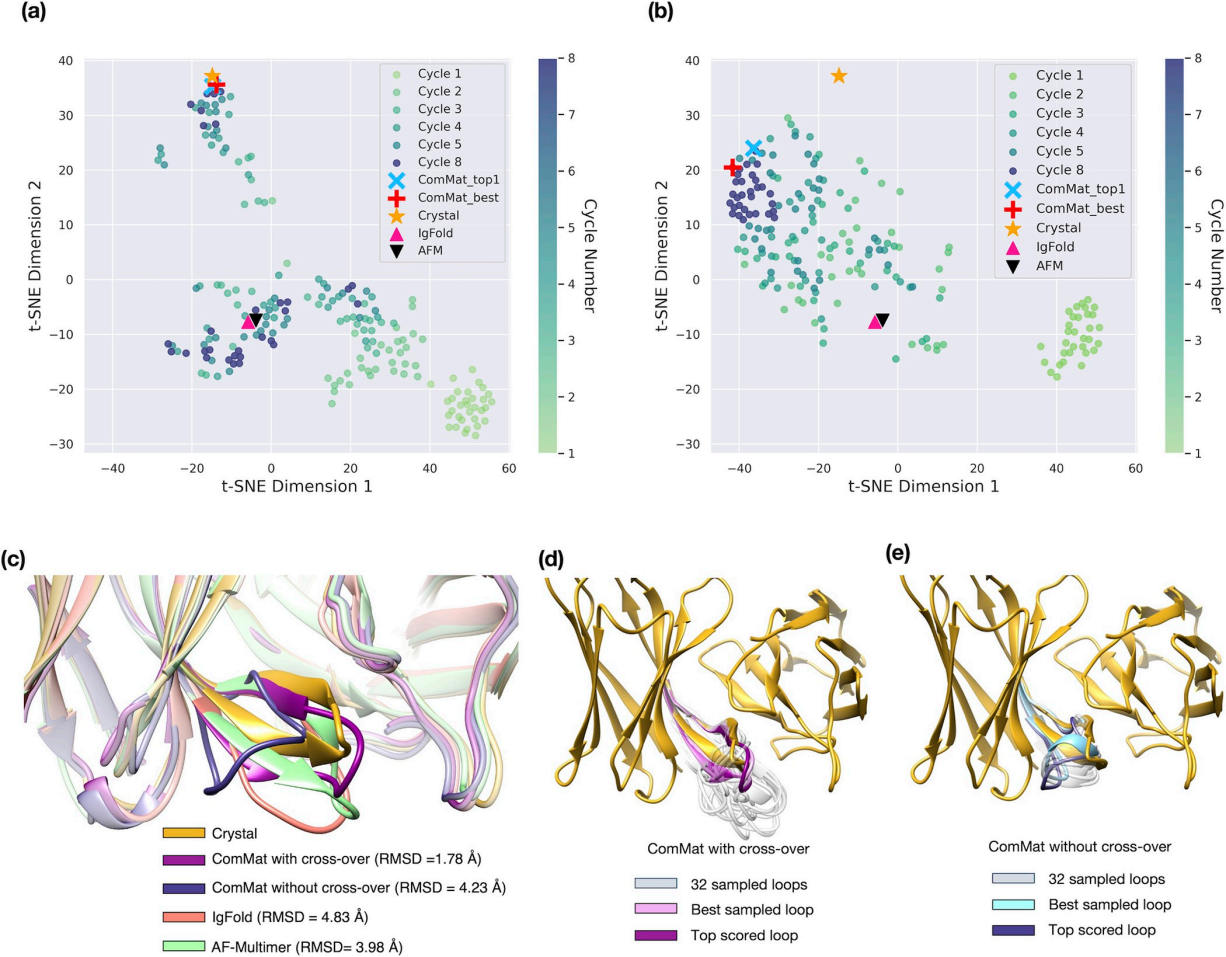
t-SNE visualization of the loop structure sampling trajectory for the Glucosyltransferase domain of Clostridium difficile toxin B binding antibody (PDB ID 7SO5, H3 loop length = 13, maximum sequence identity to the training set = 38.5%) by (a) a single-inference sampling with ComMat trained with cross-over (*N* = 32) and (b) 32 independent sampling with ComMat without cross-over. The structures obtained through the eight cycles of ComMat are indicated with round dots. Crystal structures, the best sampled and scored models by ComMat, and predictions by IgFold and AF-Multimer are denoted with different symbols. Comparison of the H3 loop structures is presented in (c), (d), and (e).

**Fig 5:**
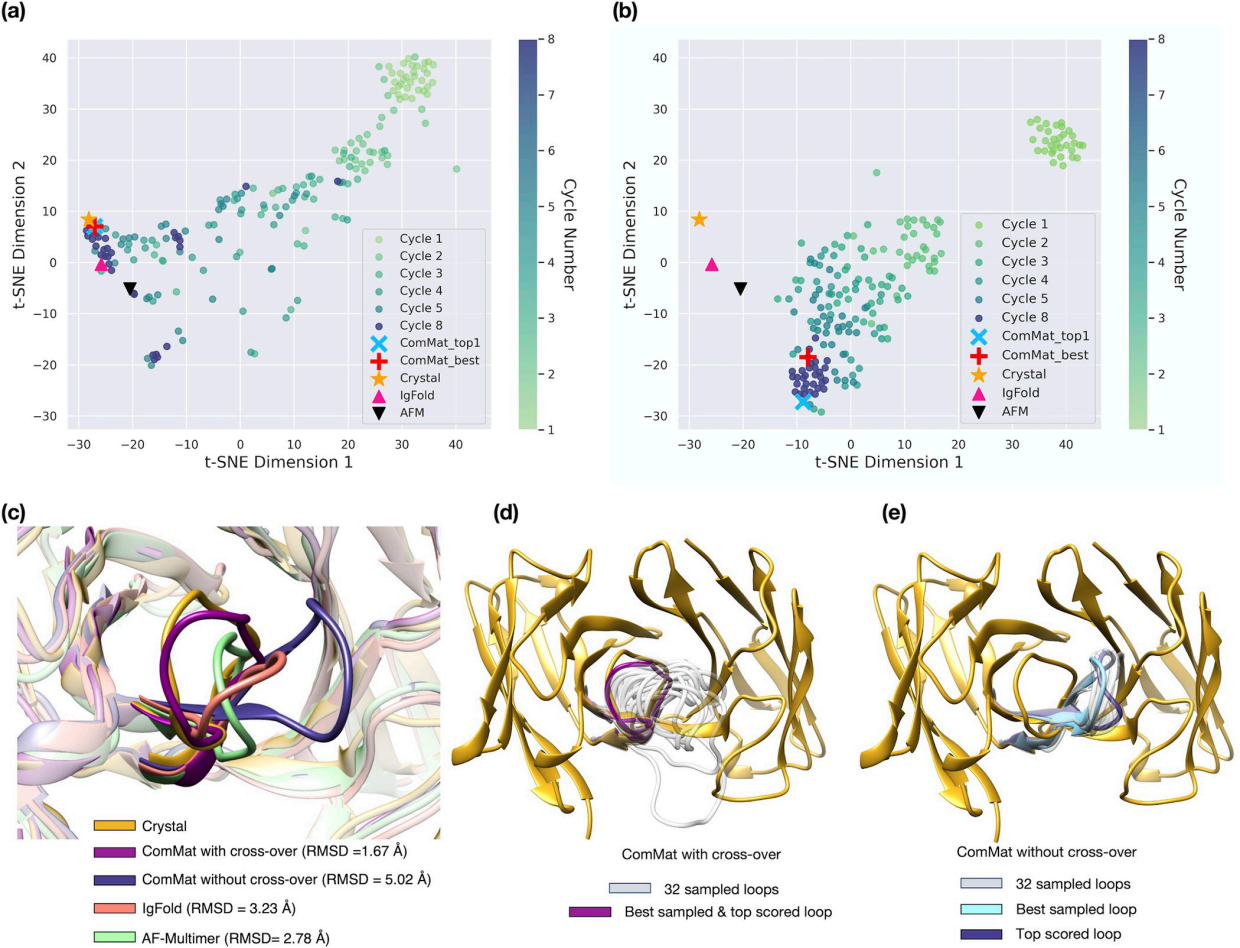
t-SNE visualization of the loop structure sampling trajectory for the SARS-Cov2 binding antibody (PDB ID 7SN1, H3 loop length = 14, maximum sequence identity to the training set = 50.0%) by (a) a single-inference sampling with ComMat trained with cross-over (*N* = 32) and (b) 32 independent sampling with ComMat without cross-over. The structures obtained through the eight cycles of ComMat are indicated with round dots. Crystal structures, the best sampled and scored models by ComMat, and predictions by IgFold and AF-Multimer are denoted with different symbols. Comparison of the H3 loop structures is presented in (c), (d), and (e).

To gain a deeper insight into how ComMat explores the loop conformational space, we closely examined the sampling trajectory through the eight cycles of ComMat for two cases illustrated in **Figs 4** and **5**, both with and without cross-over for *N* = 32, as previously outlined.

In (a) and (b) of **Figs 4** and **5**, the loop samples are depicted in a two-dimensional space that best represents the true loop RMSD distances among the sampled structures using t-SNE plots. In **Fig 4** (and Fig **5**), the ComMat cycle began with light green dots positioned near the lower right (and upper right) corners of (a) and (b). As the ComMat cycle progressed, the sampling trajectory of the ComMat model with cross-over (*N* = 32) in **Figs 4A** and **5A** diversified into various endpoints, observed as scattered dark blue dots. Some endpoints approached the ground-truth crystal structures. However, the sampling trajectory obtained by 32 independent runs of ComMat without cross-over, shown in **Figs 4B** and **5B**, led to rather convergent endpoints, represented by the clustered dark blue dots, which were distant from the ground truth crystal structure.

Structures of the best sampled and top scored loops by ComMat, alongside those predicted by IgFold and AF-Multimer, are compared in **Figs 4C** and **5C**. **Figs 4d** and **5D** display the endpoint structures sampled by ComMat with cross-over, while **Figs 4E** and **5E** show the endpoint structures sampled by ComMat without cross-over.

Although the overall area in the two-dimensional space covered by ComMat with cross-over was not larger than that by ComMat without cross-over, effective structural sampling was achieved by community maturation. The ComMat architecture with cross-over facilitated effective coverage of the structural space by diversifying its endpoint samples. This robust feature of broader exploration of the structural space could potentially enhance the accuracy of structure prediction further when combined with an effective scoring model. Moreover, proteins exhibiting multiple conformational states may be effectively sampled using the community maturation concept introduced here. This sampling approach might also be extended to design proteins with diverse conformations.

### 2.5. Dependency of the Sampling Performance on the Community Size of ComMat

We conducted an analysis to assess the impact of the community size (*N*) on the performance of ComMat. The overall performance demonstrates an increase with the community size, as evidenced in **S1 Table**. Notably, the most substantial improvement in sampling performance occurs from *N* = 1 (corresponding to no cross-over) to *N* = 2. This transition shows a decrease in median RMSD of the best sampled loops from 2.29 Å to 1.84 Å and an increase in the sampling rate within < 2 Å from 46.1% to 54.0% on the IgFold set, when generating the same number [32] of loop structures. However, the performance improvement from *N* = 2 to *N* = 32 exhibits a more moderate trend, reaching a median RMSD of 1.62 Å and a success rate of sampling < 2 Å at 60.9% with *N* = 32.

**Fig 6:**
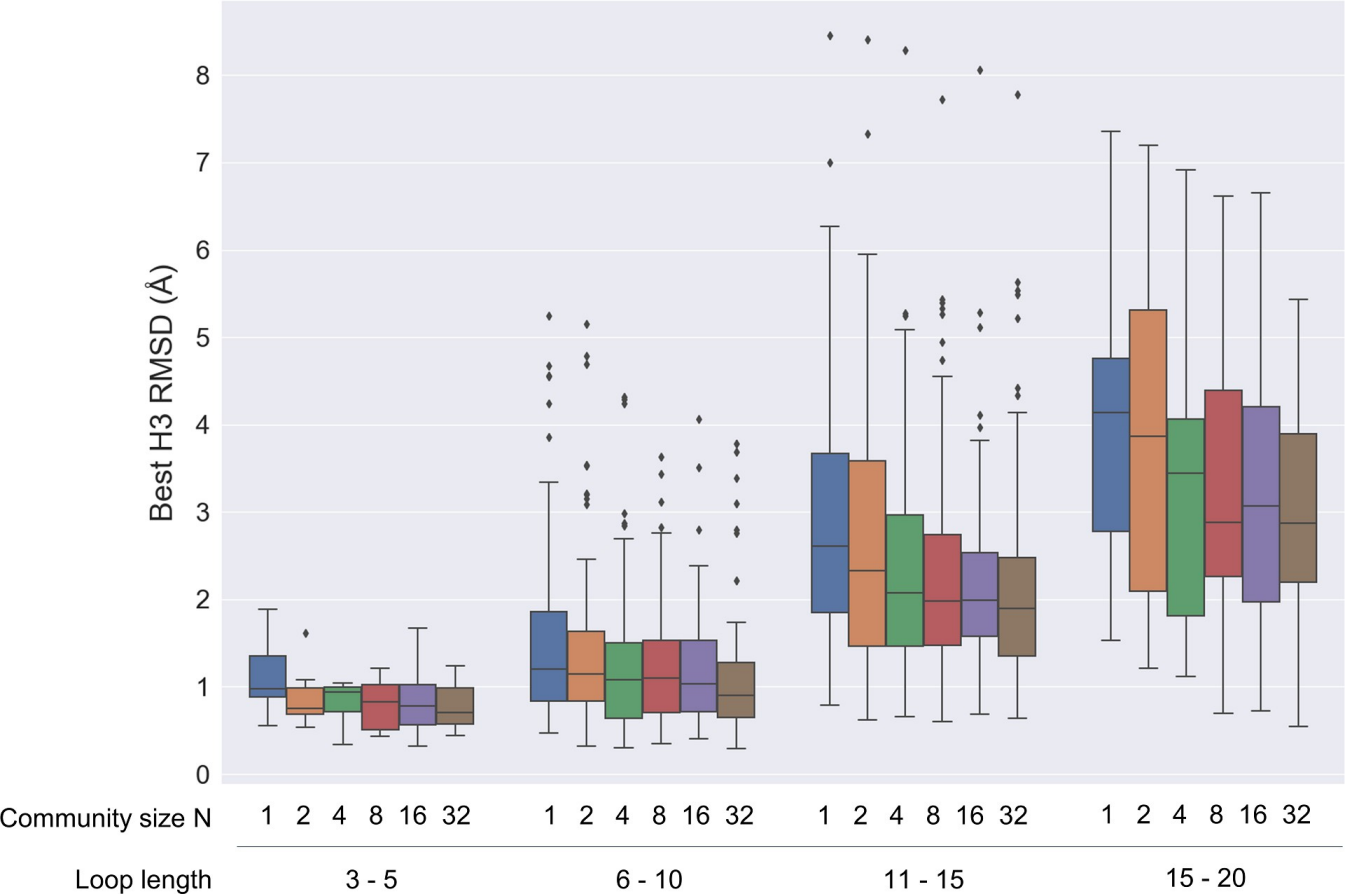
Dependency of RMSD of the best sampled loops on the community size for different loop length ranges.

The performance dependence on the community size might vary depending on the complexity of the problem at hand. Therefore, we examined the dependence on the CDR H3 loop length, as presented in **Fig 6**. The source data is provided in **S3 Table**. Across all examined loop length ranges, there is a tendency for sampling performance to enhance with an increase in the community size. Notably, the improvement is more pronounced for longer loops with larger community sizes, indicating the potential for more extensive sampling with a larger community.

The community maturation integrated into the ComMat model, facilitating information exchange among community members, could potentially be extended to other domains of structure prediction and design problems. This study serves as a foundation for further advancements by refining the architecture components, initialization, cross-over, and pair feature update tailored to specific problems.

## 3. Materials and methods

### 3.1. Preparation of the Dataset for Training, Validating, and Testing ComMat

Given the limited availability of antibody structures, our training dataset was expanded to include non-antibody protein interface loops, in addition to antibody and nanobody loops with or without bound proteins. For the validation set, we utilized the RosettaAntibody benchmark set (47 targets) [28], previously employed to evaluate antibody structure prediction methods. For the test set, we employed the antibody benchmark set (197 targets, published after July 1, 2021), previously designed for testing the IgFold model [19].

Construction of the training set involved the collection of antibody structures from the RCSB Protein Data Bank (PDB) [29] as of June 30, 2021. Those bound to non-protein antigens were excluded. To eliminate redundancy with the validation set, we clustered the resulting antibodies using a CDR H3 loop sequence identity cutoff of 70%, resulting in 2,027 clusters. Clusters containing at least one validation target were excluded. This process yielded a final set of 1,981 clusters comprising 8,502 structures.

Additionally, an additional dataset of non-antibody protein interface loops was curated. This dataset comprised loops located at protein dimer interfaces with a resolution of 3.0 Å or better, gathered from the RCSB Protein Data Bank as of June 30, 2020. Redundancies were eliminated by selecting representative structures with no more than 40% protein sequence identity and 70% loop sequence identity. Loop residues were identified based on Pross’s secondary structure categorization [30]. The selected loops consisted of between 5 and 15 standard amino acids at the protein-protein interface, with at least five residues interacting with its partner protein at C_β_ (C_α_ for GLY) distances of < 8 Å. This process resulted in a non-redundant set of 3,382 non-antibody protein interface loops. The effect of including general protein loops in the training data was not substantial, leading to a slight improvement in sampling performance; the median loop RMSD of the best-sampled loops decreased to 1.63 Å from 1.68 Å.

The complete list of training loops, encompassing both antibody and non-antibody protein loops, can be found at https://github.com/seoklab/ComMat.

### 3.2. ComMat implementation

The ComMat algorithm is presented as pseudocode in **S1 Fig**, and the model parameters are listed in **S2 Table**, containing 2.7 M trainable parameters. **Fig 1** in the main text illustrates the overall flow of ComMat, encompassing initialization, cross-over, and Z-update modules, further elaborated below. Following eight iterative cycles, ComMat predicts backbone and side chain torsion angles from the final single features to construct full structures. Additionally, predicted LDDT values, derived from single features, serve as a quantitative metric to assess the accuracy of the sampled structures. The source code implementation of the ComMat algorithm is available at https://github.com/seoklab/ComMat.

#### 3.2.1. Initialization

The sequence embedding of ESM-2 [31], a pretrained protein language model, is utilized to generate initial single features ***S***^(0)^. These features are identical for all community members.

Initial loop structures are generated by evenly spacing the loop residue gases between stem residues. Subsequently, structure features ***T***^(0)^ are created through translational and rotational perturbations to the residue gases. Weak perturbations are also applied to non-loop residues, expecting robust performance in real-world scenarios.

Initial pair features **Z**^(0)^ are derived by incorporating the distogram features from initial structures, concatenated single features (row-wise and column-wise), and chain/loop information.

#### 3.2.2. Cross-over

Prior to cross-over among community members, single features ***S***^(*k*)^ are updated to ***S***′^(*k*)^ using the IPA architecture of AlphaFold [1], integrating information from the pair and structure features (Refer to **Fig 1**).

The updated single features ***S***′^(*k*)^ undergo cross-over using community-wide attention, resulting in ***S***^+(*k*)^, as depicted in **Fig 1**. The process involves deriving query, key, and value parameters (*q*_*ni*_, *k*_*ni*_, *v*_*ni*_) from a single feature projection (*s*_*ni*_) for the *i*th residue of the *n*th member as

$$q_{ni},k_{ni},v_{ni}=LinearNoBias(s_{ni}).$$

These parameters facilitate calculating attention values between community members, enabling comprehensive information exchange within the community.


$$a_{nmi}={softmax}_{m}\left(\frac{1}{\sqrt{c}}{q_{ni}}^{T}k_{ni}\right)$$

$$o_{ni}=\sum_{m}a_{nmi}\;v_{mi}$$

$$s_{ni}^{+}={Linear(concat(o}_{ni}))$$

#### 3.2.3. Z-update

The pair features undergo an update using the updated single features and structure features, followed by a triangular update, as illustrated in **Fig 1**. The triangular update involves two blocks of triangular multiplicative update and triangular self-attention, inspired by the Evoformer module of AlphaFold [1], which ensures proper reflection of protein geometric restraints.

### 3.3. ComMat training

The training losses and the data augmentation strategies are explained below. Owing to memory limitations, 100 nearest residues were cropped based on the center of the two stem residues of the loop of interest during training.

#### 3.3.1. Training loss

The loss function of ComMat *L* combines the minimum structural loss $L_{i}^{structure}$ among community members with the mean of auxiliary loss $L_{i}^{aux}$. The structural loss comprises FAPE, distogram, and torsion losses of AlphaFold [1], along with loop RMSD loss. The auxiliary loss comprises predicted LDDT loss and structural violation loss from AlphaFold. This approach aims to optimize sampling performance while also ensuring reliable accuracy estimation and maintaining high structural fidelity.


$$L=\min\left(L_{i}^{structure}\right)+mean(L_{i}^{aux})$$

$$L_{i}^{structure}={1.0\;L}_{i}^{loopRMSD}+{1.0\;L}_{i}^{FAPE}+0.3\;L_{i}^{distogram}+0.5\;L_{i}^{torsion}$$

$$L_{i}^{aux}={0.01\;L}_{i}^{pLDDT}+{1.0\;L}_{i}^{violation}$$

#### 3.3.2. Data augmentation

Various approaches were employed for data augmentation. Random translational perturbations were applied to the cropping center, limited to a maximum magnitude of 2 Å. For antibodies bound to antigens, antigen was removed with a 70% probability. Additionally, for antibodies, other CDR loops besides H3 were included with a 50% probability.

### 3.4. ComMat geometry optimization

The final structure generated by ComMat underwent geometry optimization after the predicted H3 loop structure was reinserted into the initial framework. Local energy optimization using the GALAXY energy function [18] was performed for each antibody structure. This optimization improved the counts of unphysical components: cis amide bonds (cis-proline) decreased from 0.61 to 0, non-planar amide bonds from 0.28 to 0.14, and van der Waals clashes from 24.6 to 0.15, as measured using TopModel [32]. The counts of D-amino acid chiral centers remained at 0.

### 3.5. Running compared methods

All compared methods, including IgFold, ImmuneBuilder, EquiFold, RosettaAntibody, and AlphaFold-Multimer 2.2 and 2.3, were run using the publicly accessible versions. Experimental structures deposited after July 1, 2021, were excluded from the template list. For the sampling test of AlphaFold-Multimer 2.2 and 2.3 reported in **Table 1**, *N* = 1 and 32 structures were generated with model 1 by using different random seeds. For prediction tests, five models of AlphaFold-Multimer 2.2 and 2.3 were employed. For IgFold and ImmuneBuilder, the codes were modified to report structures generated by all four models with different parameters, and versions that include structure refinement were used for performance measurement. EquiFold generates a single structure, so no modifications were made. For RosettaAntibody runs, 2,800 structures were generated according to the original protocol [27], and *N* = 1 and 32 structures were selected based on Rosetta energy.

## Supporting information

S1 Table Dependence of ComMat performance on the community size. (XLSX)

S2 Table List of ComMat model parameters. (XLSX)

S3 Table Performance of unrefined ComMat with different community sizes for the individual targets of IgFold test set. (XLSX)

S4 Table Information about each pdb in the IgFold test set. (XLSX)

S5 Table Performance of refined ComMat and other antibody loop modeling methods. (XLSX)

S6 Table Sampling performance of refined ComMat and other antibody loop modeling methods for the individual targets of IgFold test set. (XLSX)

S7 Table Top scored performance of refined ComMat and other antibody loop modeling methods for the individual targets of IgFold test set. (XLSX)

S1 Fig A pseudocode for the ComMat workflow. (TIF)
