# Supplementary figures and images for: Protein loop structure prediction by community-based deep learning and its application to antibody CDR H3 loop modeling
Source: PLoS Comput Biol. 2024 Jun 24;20(6):e1012239. doi: 10.1371/journal.pcbi.1012239 (PMC11226077; doi:10.1371/journal.pcbi.1012239)

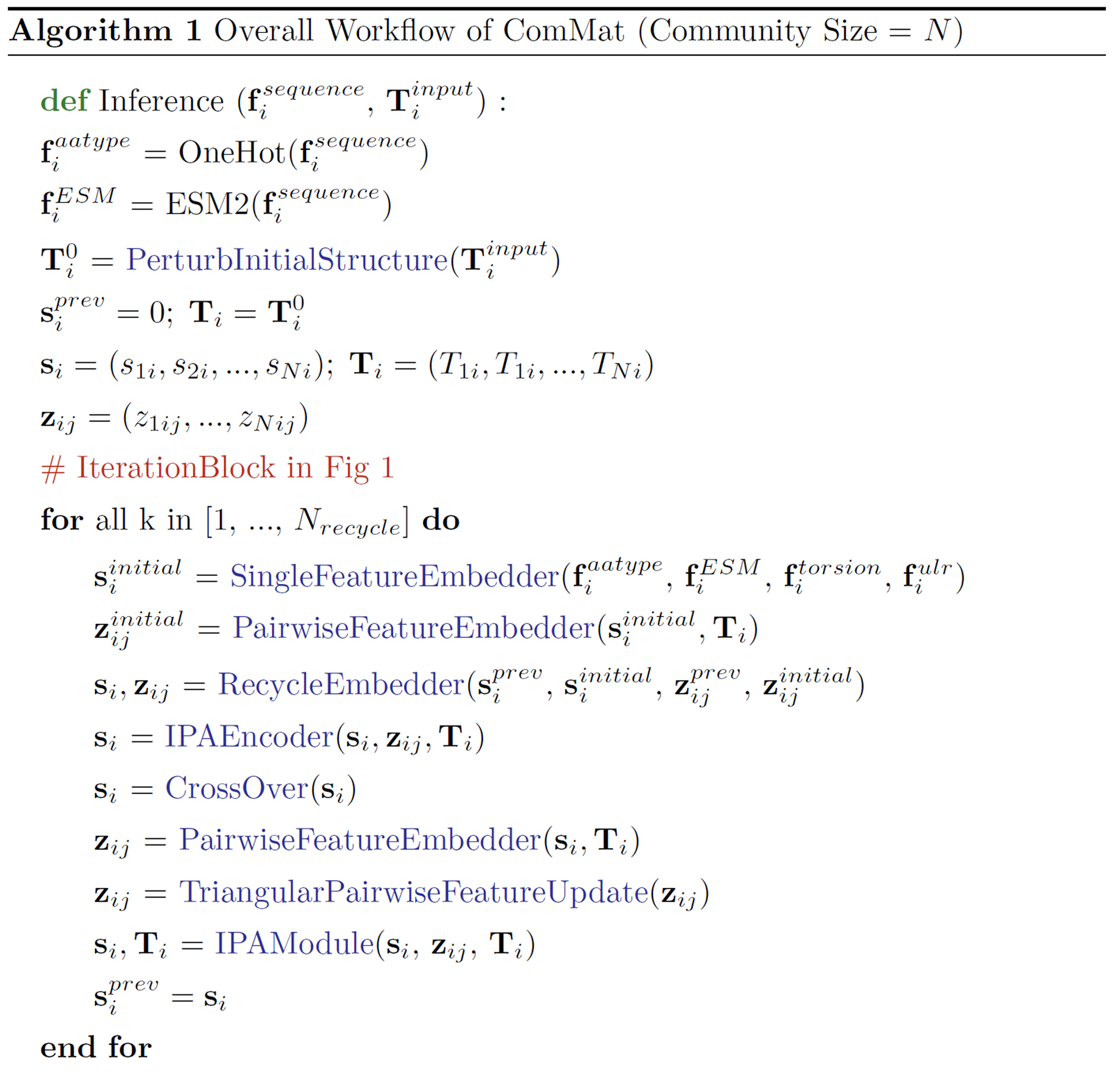

Supplement: S1 Fig — (TIF) [file pcbi.1012239.s008.tif]
